# Assessment of transcriptional importance of cell line-specific features based on GTRD and FANTOM5 data

**DOI:** 10.1371/journal.pone.0243332

**Published:** 2020-12-21

**Authors:** Ruslan N. Sharipov, Yury V. Kondrakhin, Anna S. Ryabova, Ivan S. Yevshin, Fedor A. Kolpakov

**Affiliations:** 1 Laboratory of Bioinformatics, Federal Research Center for Information and Computational Technologies, Novosibirsk, Russian Federation; 2 Specialized Educational Scientific Center, Novosibirsk State University, Novosibirsk, Russian Federation; 3 BIOSOFT.RU, Ltd, Novosibirsk, Russian Federation; Università degli Studi di Milano, ITALY

## Abstract

Creating a complete picture of the regulation of transcription seems to be an urgent task of modern biology. Regulation of transcription is a complex process carried out by transcription factors (TFs) and auxiliary proteins. Over the past decade, ChIP-Seq has become the most common experimental technology studying genome-wide interactions between TFs and DNA. We assessed the transcriptional significance of cell line-specific features using regression analysis of ChIP-Seq datasets from the GTRD database and transcriptional start site (TSS) activities from the FANTOM5 expression atlas. For this purpose, we initially generated a large number of features that were defined as the presence or absence of TFs in different promoter regions around TSSs. Using feature selection and regression analysis, we identified sets of the most important TFs that affect expression activity of TSSs in human cell lines such as HepG2, K562 and HEK293. We demonstrated that some TFs can be classified as repressors and activators depending on their location relative to TSS.

## Introduction

The identification of complex mechanisms of regulation of gene expression in higher eukaryotes is a major challenge for modern computational biology. The key question is to better understand the role of transcription factors (TFs), which regulate the transcriptional machinery in cells. Over the past decade, ChIP-Seq has become the most popular experimental technology for studying the genome-wide interactions between TFs and DNA. To date, several databases, such as GTRD (http://gtrd.biouml.org/) [1, 2], ENCODE (https://www.encodeproject.org/) [3], ChIP-Atlas (https://chip-atlas.org/) [4], and ReMap (http://tagc.univ-mrs.fr/remap/) [5] have been created to systematically process and collect ChIP-Seq datasets obtained by applying different peak callers to the primary ChIP-Seq data.

To study the effect of TF binding on gene expression, it is common practice to analyze the integrated ChIP-Seq and RNA-Seq data [6, 7], since RNA sequencing is a source of transcription level data. Another source of experimental data on the level of transcription is the CAGE (Cap Analysis of Gene Expression) technology. Thus, FANTOM5 (fifth edition of the FANTOM database) contains profiled TSSs in the human genome using CAGE with single-molecule sequencers (HeliScope) and a generated atlas of CAGE expression levels (TSS activities) in primary cells, tissues and cell lines [8]. Initially, the GRCh37/hg19 assembly was used as the reference human genome. This atlas was later redesigned to fit newer genome assembly–GRCh38/hg38 [9].

The aim of our study was to assess the direct influence of TF binding on activities of TSSs in most of the studied human cell lines. For this purpose, we initially generated a large number of features that were defined as the presence or absence of TFs in the different promoter regions around each available TSS. To generate features, we used the ChIP-Seq datasets of human TF binding regions (TFBRs) collected in the GTRD database and TSS activities from the FANTOM5 atlas. For further selection of the most important features, we used the stepwise forward regression where the selection of features was carried out by an automatic stepwise procedure. As a result, the constructed regression models made it possible to compose narrow lists of TFs, which had significant influence on TSS activities in the considered cell lines. In other words, the composed lists consisted of features that directly related with TSS activity.

Finally, it is important to note that efforts to create atlases of candidate cis-regulatory elements (promoters, enhancers, silencers, insulators) of human and mammalian genomes has been increased over the past decade [10–19]. A breakthrough in high-throughput sequencing technologies [20], which made it possible to analyze the genomic landscape and gene expression from different points of view, as well as large amounts of data obtained for various types of cells and activation stimuli, made it possible to approach the creation of such atlases for the most studied taxa, human and mouse. Nevertheless, due to the extreme complexity (a wide variety of types of primary cells and cell lines; cell-specific functions of enhancers [21]; features of gene expression in various cells; differences in the implementation of the cell program depending on an external or internal stimuli, etc.), the solution of this problem is far from complete. Most of the research has focused on gene expression activators such as enhancers, while the regions that suppress gene expression–silencers–are poorly understood [22].

## Materials and methods

**Fig 1:**
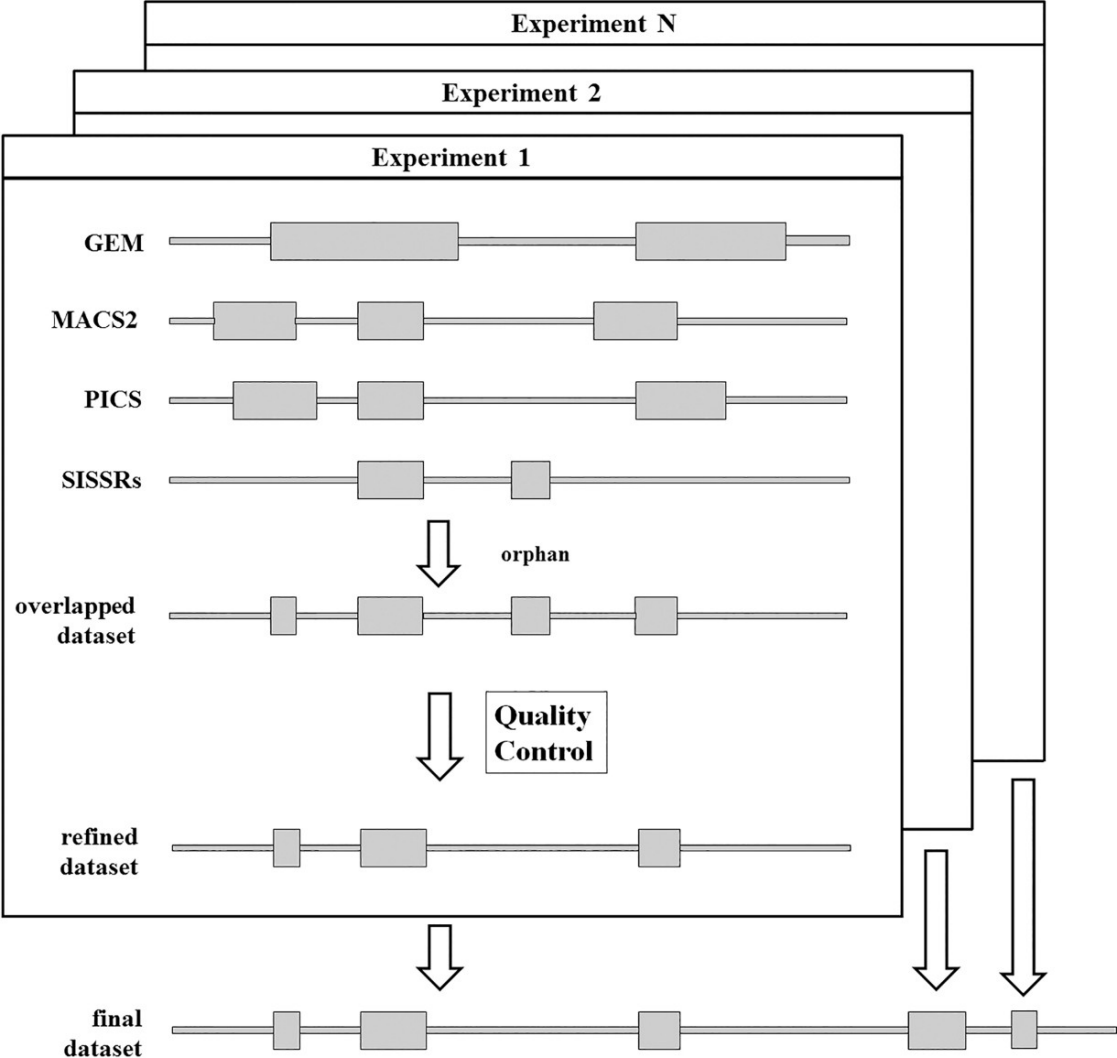
The workflow of meta-processing the ChIP-Seq datasets.

In general, the key datasets for our study were the overlapped sets of TFBRs that were compiled through a three-step meta-processing of the ChIP-Seq datasets collected in the GTRD database. Thus, for a given cell line and a given TF, we initially selected only those ChIP-Seq experiments in which the cell line was not treated. In the first step of meta-processing, the following peak callers were applied to the same raw data obtained from individual ChIP-Seq experiment: GEM [23], MACS2 [24], PICS [25], and SISSRs [26], see Fig 1. In the second step, four resulting sets of TFBRs were overlapped and the False Positive Control Metric (FPCM) [27] was applied to perform quality control for the overlapped dataset.

If FPCM exceeded the pre-specified threshold value of 3.0, then all so-called orphans (such TFBRs that did not overlap with other initial TFBRs) were removed from the overlapped dataset. Thus, the single refined dataset was identified for data from the given ChIP-Seq experiment. Finally, in the third step, the single final dataset was obtained for the given TF as a union of all the refined datasets corresponding to distinct experiments.

To determine the primary set of the regression features (say, PRIMARY_FEATURES), we initially defined the following eight promoter regions (in base pairs) around each available TSS:

$$\begin{array}{l} \left[‐5000,\;‐1001\right],\;\left[‐1000,\;‐501\right],\;\left[‐500,\;‐201\right],\;[‐200,\;‐101],\;\\ \;\left[‐100,\;0\right],\;\left[1,\;100\right],\;\left[101,\;500\right]\;\mathrm{and}\;\left[501,\;1000\right]. \end{array}$$ (1)

The genomic coordinates of 209,911 TSSs and their activities were extracted from the FANTOM5 atlas [9]. The first eight real-valued features were defined as relative numbers of TFs that bonded (at least, partially) these promoter regions. In detail, if m different TFs were available for a given cell line and TFBRs of m_0_ TFs overlapped with the [x_1_, x_2_]-promoter region, then the feature Abundance[x1, x2] was determined as the ratio m_0_ / m. In other words, the feature Abundance[x_1_, x_2_] is an estimate of the concentration of TFBRs within a given [x_1_, x_2_]-promoter region. According to its definition, each feature Abundance[x_1_, x_2_] varies in the range [0, 1]. In general, these features indicate the abundances of promoter regions with TFBRs. It is important to note that these Abundance-features can be interpreted as indicators of cis-regulatory modules. Indeed, according to their definitions, cis-regulatory modules represent the stretches of DNA, where a number of TFs can bind and regulate the expression of nearby genes and regulate the rate of their transcription [28]. The next features were binary. Each binary feature took values {1, 0} depending on the presence or absence of TFBRs of individual TFs in a given promoter region. Thus, PRIMARY_FEATURES consisted of 8×(m+1) features. One can expect that considerable number of the primary features in PRIMARY_FEATURES may be irrelevant in particular regression models. In general, if there are hundreds or even thousands of features, then it is advisable to perform feature selection to create a regression model that includes only the most important features. For this purpose, we used well-known stepwise forward regression approach. According to this approach, we selected at each step the single feature from PRIMARY_FEATURES the inclusion of which into ordinary least squares regression gave the highest correlation (say, *R*_o-p_) between the predicted and observed transcriptional activity.

Finally, it is important to note that we have used all of the available 209,911 TSSs, although one might expect some of them to be falsely generated due to CAGE technology. For such TSSs, regression models must correctly predict negligible (or almost negligible) expression levels due to the binarity of features. Indeed, falsely generated TSSs are not transcriptionally active, therefore, promoter regions around such TSSs should not contain TFBRs. According to the definition of our features, at least the majority of features for such TSSs have to take zero values. In turn, for our regression analyses, we used only linear regression models. Therefore, levels of expression are predicted as the inner products of regression coefficients and zero-valued features. As a result, these products also have zero values (or near zero-values).

## Results and discussion

### Primary regression models

Basically, we focused on the following three human cell lines: HepG2 (hepatoblastoma), K562 (myelogenous leukemia), and HEK293 (embryonic kidney). We selected these cell lines because they were the most representative cell lines in GTRD. Thus, HepG2 was represented by 230 initial ChIP-Seq datasets obtained for 169 TFs (see Table 1); HEK293 was represented by 210 datasets for 177 TFs, and 304 datasets for 186 TFs were available for K562.

**Table 1:** Summary on ChIP-Seq datasets.

Cell line	Number of initial ChIP-Seq datasets	Number of distinct TFs	Number of features
HepG2	230	169	1360
K562	304	186	1496
HEK293	210	177	1424

PRIMARY_FEATURES sets were generated as described in the Materials and Methods for each cell line independently. Thus, PRIMARY_FEATURES for HepG2 consisted of 1360 (= 8 × 170) features that represented the presence/absence of TFBS in the promoter regions defined in (1).

The stepwise forward regression was applied to the composed PRIMARY_FEATURES to select the most important features and obtain a primary regression model. This regression described the relationship between TSS activities and the 20 most important features. The log-transformed expression levels (say, LTE-levels) from the FANTOM5 atlas were used as TSS activities hereinafter. For a given expression level EL, the LTE-level was defined as {0, if EL < 2; lg(EL) otherwise}.

Table 2 contains the primary regression model obtained for the HepG2 cell line. S1 and S2 Tables contain the primary regression models obtained for K562 and HEK293, respectively. All the most important features selected by stepwise forward regression were sorted in the order of their selection. The accuracy of each intermediate regression model was measured by the Pearson correlation coefficient *R*_o-p_ between the predicted and observed transcriptional activities. The values of *R*_o-p_ demonstrated that it was sufficient to implement only 20 steps of stepwise forward regression, since increments of *R*_o-p_ in the last steps became almost negligible, see Table 2. All selected features turned out to be statistically significant, *p*-value < 10^−67^.

**Table 2:** Primary regression model for the HepG2 cell line.

Feature	Correlation coefficient, *R*_o-p_	Increment of correlation coefficient	Regression coefficient	*p*-value
Abundance [-100, 0]	0.585	0.585	0.261	7.734 × 10^−68^
TAF1 [1, 100]	0.650	0.135	0.329	< 1.0 × 10^−300^
HEY1 [1, 100]	0.677	0.027	0.304	< 1.0 × 10^−300^
NONO [1, 100]	0.689	0.012	0.292	< 1.0 × 10^−300^
HEY1 [501, 1000]	0.698	0.009	0.243	< 1.0 × 10^−300^
JARID1A [101, 500]	0.703	0.005	0.239	< 1.0 × 10^−300^
C/EBP δ [1, 100]	0.706	0.003	0.147	3.185 × 10^−291^
p53 [-1000, -501]	0.709	0.003	0.479	3.646 × 10^−126^
KLF10 [-100, 0]	0.711	0.002	0.113	4.117 × 10^−100^
TBP [-100, 0]	0.713	0.002	0.139	4.066 × 10^−226^
KLF10 [501, 1000]	0.715	0.002	-0.208	1.653 × 10^−166^
HEY1 [-100, 0]	0.717	0.002	-0.141	< 1.0 × 10^−300^
HEY1 [-500, -201]	0.719	0.002	0.133	< 1.0 × 10^−300^
TBP [101, 500]	0.721	0.002	-0.097	5.945 × 10^−111^
TAF1 [-100, 0]	0.722	0.001	0.116	2.359 × 10^−136^
Sp1 [-200, -101]	0.723	0.001	0.126	2.968 × 10^−157^
ELF1 [-100, 0]	0.724	0.001	0.107	5.219 × 10^−122^
p53 [501, 1000]	0.725	0.001	0.492	3.496 × 10^−131^
MYC [-100, 0]	0.726	0.001	0.124	1.053 × 10^−102^
Sp1 [101, 500]	0.726	< 0.001	-0.148	4.647 × 10^−111^

In general, the accuracy of the primary regression models turned out to be quite acceptable, since *R*_o-p_ varied in the range [0.626, 0.726], see Table 3. To assess the reliability of regression models we cross-validated them. For this purpose, we have split at random the entire set of features into training and test sets of the equal size. After that, a regression model was built on the training set, and LTE-levels were predicted independently in both sets using the constructed regression model. Thus, Table 3 contains also the accuracies of primary regression models obtained on training and test sets. It turned out that the constructed regression models are quite reliable because the differences between *R*_o-p_ are negligible. In other words, the regression models were not overfitted. The regression coefficients were obtained using the ordinary least square regression, which was built in step 20.

**Table 3:** Accuracies of the primary regression models measured by the *R*_o-p_ correlation coefficient.

Cell line	Entire set of features	Training set	Test set
HepG2	0.726	0.725	0.727
K562	0.704	0.706	0.702
HEK293	0.626	0.625	0.624

The sign of the regression coefficient may clarify the function of some TFs. If the coefficient is positive, then TF can be classified as a transcription activator or coactivator. If the coefficient is negative, then TF can be classified as a repressor or corepressor. According to Table 2, some TFs can act as activator and repressor depending on location of the binding site with respect to TSSs. For example, HEY1 acted as an activator in the three promoter regions [1, 100], [501, 1000], [-500, -201], while it acted as a repressor in the [-100, 0]-promoter region. It is important to note that our regression model re-revealed this well-known role for HEY1 [29]. It is important to note that HEY1 prefer to act as activator for some genes and as repressor for other genes. In particular, HEY1 preferred to avoid binding to both [-100, 0] and [1, 100]-promoter regions simultaneously. In order to confirm this avoidance, we calculated the ratio of the observed and estimated probabilities of simultaneous binding to these promoter regions. It turned out that the ratio was equal to 0.543, hence the observed simultaneous binding is essentially rare than can be expected. Table 2 also demonstrates the same effect for KLF10. It can be classified as an activator if it is located in the [-100, 0]-promoter region, while it acts as a repressor in the [501, 1000]-promoter region. Our regression model once again confirmed the well-known fact that KLF10 is a repressor of multiple genes in many cell types [30]. Finally, SMAD5, JARID1B and MLL can be classified as activators and repressors in K562 or HEK293 (see S1 and S2 Tables) depending on their location.

It is important to note that the influence of TF binding on TSS activity in the K562 cell line was also studied [31] using data from the ENCODE consortium [3]. TSS activities were represented by CAGE expression levels, and approximately 120 ChIP-Seq datasets were used in this study. A single feature for given TSS and TF was defined as the average number of ChIP-Seq reads within the [-50, 50] region of the promoter. As a result, a list (say, List-40) of 40 the most important TFs (features) was identified by random forest regression model. It is difficult to compare the results directly because of the differences of feature and regression types. To overcome this difficulty, *we* extracted 320 *(8 ×* 40) binary features from PRIMARY_FEATURES, which represented TFs in List-40 and applied stepwise forward regression to them. The resulting primary regression model (say, K562_List-40) is available as S3 Table. Comparison of K562_List-40 and our primary model in S1 Table indicated that the sets of selected features are quite different. Thus, only three (15%) features, namely, NF-YA [-100, 0], Sp1 [-200, -100] and SIX5 [-100, 0], were represented in both models. It seems likely that the features in S1 Table are more preferable and more reliable than the features selected by K562_List-40, since the accuracy of the primary regression in Table 2 (*R*_o-p_ = 0.704) is significantly higher than the accuracy of K562_List-40 (*R*_o-p_ = 0.617).

### Comparative analysis of cell lines

To increase the accuracy of regression models it is necessary to generate additional features and involve them in regression models. For this purpose, we performed a comparative analysis of cell lines using their transcription activity profiles. We determined the transcription activity profile for the given cell line as a set of 209,911 TSS activities from the FANTOM5 expression atlas.

**Fig 2:**
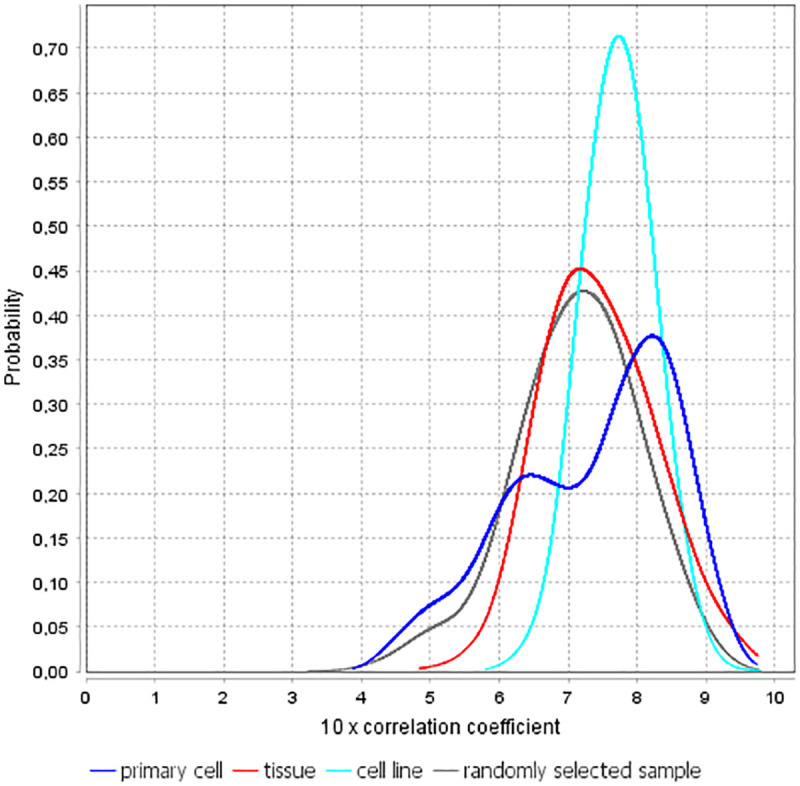
Empirical densities of the Pearson correlation coefficient between objects in the FANTOM5 atlas.

In general, this atlas contains expression levels for the following three types of objects: cell line, primary cell, and tissue. We analyzed the similarity of objects of the same type using correlations between their transcription activity profiles. In addition, we considered a randomly selected sample to control the similarities between objects of various types. It turned out that there was a high correlation between the considered objects, see Fig 2. Moreover, the highest correlations were observed between different cell lines (see Table 4). It is important to note that RNA-Seq data also confirmed similarity between cell lines. To demonstrate this, we calculated correlations between 25 distinct cell lines. For this purpose, we used the RNA-Seq datasets generated by the ENCODE^3^ consortium. It turned out that correlation coefficient varied in the range [0.423, 0.845], and mean correlation was equal to 0.681, when protein-coding transcripts from Ensembl were used for calculation of correlation.

**Table 4:** Summary on correlations between objects in the FANTOM5 atlas.

Type of objects in the FANTOM5 atlas	Size	Mean correlation	Minimal correlation	Maximal correlation
primary cell	432	0.735	0.389	0.974
tissue	135	0.744	0.485	0.975
cell line	241	0.772	0.581	0.978
randomly selected sample	300	0.712	0.322	0.975

Obviously, from a biological point of view, it is not surprising that there are relationships between primary cells, or/and tissues, or/and cell lines, because tissues are composed of different types of primary cells, and cell lines are immortalized or cancer-transformed cells that resemble their tissue of origin [32]. In other words, one can expect that many pairs of primary cells, tissues, and cell lines can be similar in terms of their transcriptional activity. However, Table 4 and Fig 2 allowed not only confirming this fact, but also estimating the strength of these relationships from a statistical point of view.

Due to the revealed similarities, it is not difficult to accurately predict the transcriptional activity profile of one cell line using the profile of another cell line. In particular, the following two regression models expressed the relationship between transcriptional activity profile for HepG2 and the profile for HEK293 or the profile for K562:

$$\begin{array}{l} HepG2=0.089+0.906\times HEK293,\;correlation\;coefficient\;R_{o‐p}=0.812,\;\\ HepG2=0.148+1.054\times K562,\;R_{o‐p}=0.766. \end{array}$$

S1 Fig demonstrates these two regression models.

### Advanced regression models

Based on comparative analysis performed in the previous section, we can confidently conclude that cell lines are similar in terms of their transcription activity profiles. In other words, the activities of many TSSs are almost identical in many cell lines. To incorporate this cell line commonality into regression models, we generated a new feature called ‘mean profile’. It was defined as a set of 209,911 mean values of activities, where an individual mean activity for each TSS was determined by averaging all of its activities in cell lines available in the FANTOM5 atlas.

The accuracy of regression model was significantly improved when stepwise forward regression was applied to the combination of PRIMARY_FEATURES and the mean profile. Thus, a comparison of *R*_o-p_ values in the first row of Table 5 with *R*_o-p_ values achieved using primary regression models (see Table 3) indicated that the accuracy increased 1.23–1.48 times. However, such a regression model has a serious disadvantage, since it is completely useless for predicting the activities of novel TSSs, which are absent in the FANTOM5 atlas. To avoid this disadvantage, we generated a new feature called ‘predicted mean profile’. This feature was determined using the following two-step procedure. In the first step, the stepwise forward regression was applied three times to PRIMARY_FEATURES determined for HepG2, K562 and HEK293 independently. As a result of the first step, three predicted profiles were obtained. In the second step, the ‘predicted mean profile’ was generated by averaging three predicted profiles. Thus, ‘predicted mean profile’ was determined by applying stepwise forward regression technique to all the PRIMARY_FEATURES defined for HepG2, K562 and HEK293. Finally, the stepwise forward regression was applied to the combination of PRIMARY_FEATURES and the predicted mean profile to select the most important features and get an advanced regression model. Table 6 contains the advanced regression model obtained for the HepG2 cell line. S4 and S5 Tables contain the advanced regression models obtained for cell lines K562 and HEK293, respectively. The accuracy of advanced regression models is demonstrated in the second row of Table 5.

**Table 5:** Accuracy of advanced regression models for the HepG2, K562 and HEK293 cell lines.

Set of features	*R*_o-p_ for HepG2	*R*_o-p_ for K562	*R*_o-p_ for HEK293
PRIMARY_FEATURES and mean profile	0.895	0.882	0.925
PRIMARY_FEATURES and predicted mean profile	0.743	0.733	0.732

**Table 6:** Advanced regression model for the HepG2 cell line.

Feature	Correlation coefficient, *R*_o-p_	Increment of correlation coefficient	Regression coefficient	*p*-value
Predicted mean profile	0.701	0.701	0.712	< 1.0 × 10^−300^
HEY1 [1, 100]	0.717	0.016	0.198	< 1.0 × 10^−300^
HEY1 [501, 1000]	0.724	0.007	0.154	< 1.0 × 10^−300^
Abundance [-100,0]	0.728	0.004	0.259	1.071 × 10^−76^
TAF1 [1,100]	0.731	0.003	0.170	2.624 × 10^−275^
NONO [1, 100]	0.733	0.002	0.147	3.106 × 10^−232^
HEY1 [-1000, -501]	0.734	0.001	0.057	1.680 × 10^−54^
AhR [-100, 0]	0.735	0.001	-0.059	1.330 × 10^−108^
Sp1 [-200, -101]	0.736	0.001	0.098	3.294 × 10^−102^
TBP [-100, 0]	0.737	0.001	0.095	2.166 × 10^−115^
c-Myc [-100, 0]	0.738	0.001	0.107	2.690 × 10^−81^
HEY1 [-5000, -1001]	0.738	< 0.001	0.060	3.009 × 10^−138^
NF-YC [501, 1000]	0.739	< 0.001	-0.168	4.312 × 10^−98^
GR [-500, -201]	0.740	< 0.001	0.425	1.164 × 10^−89^
HEY1 [-100, 0]	0.740	< 0.001	-0.083	5.989 × 10^−123^
TAF1 [-100, 0]	0.741	< 0.001	0.090	9.918 × 10^−90^
TAF1 [-1000, -501]	0.741	< 0.001	-0.098	9.337 × 10^−90^
HEY1 [-500, -201]	0.742	< 0.001	0.069	1.032 × 10^−81^
HNF3G [101, 500]	0.742	< 0.001	-0.099	1.191 × 10^−88^
SSRP1 [101, 500]	0.743	< 0.001	0.112	2.267 × 10^−87^

It is important to note that the predicted mean profile was the most important feature in all three advanced regression models. Therefore, one can conclude that common (i.e. not specific to the cell line) transcription processes are dominant in different cell lines. However, cell line specificity can also be detected using advanced regression models. Thus, it is well known that HEY1 is involved in the regulation of self-renewal of liver cancer cells [33]. Therefore, it was not surprising that HEY1 was the most represented TF in the most important features for HepG2. According to Table 6, HEY1 was observed in the six most important features. In other words, its binding to 6 promoter regions [-5000, -1001], [-1000, -501], [-500, -201], [-100, 0], [1, 200] and [501, 1000] was important for transcription in the HepG2 cell line. Moreover, based on comparison of the primary and advanced regression models (see Tables 2 and 6), one can conclude that the features of the advanced regression model were more specific for cell lines than those of the primary regression model. Indeed, HEY1 was observed only in the four most important features of the primary model. Additionally, it is well-known that HNF3G (hepatocyte nuclear factor 3-gamma) plays an important role in the development, differentiation and regeneration of the liver [34]. Therefore, it was not surprising that HNF3G was selected using the advanced regression (see Table 6) but it was not selected using primary regression.

It is interesting to note that the most important features correlated with some additional features from PRIMARY_FEATURES. Thus, Table 7 contains five features that most correlate with individual most important feature identified for the HepG2 cell line. It is not difficult to see that almost all important features (excluding GR[-500, -201]) highly correlated with the corresponding Abundance-features. In particular, the correlation coefficient between HEY1[501, 1000] and Abundance[501, 1000] is equal to 0.689 while for the pair (HEY1[-1000, -501], Abundance[501, 1000]) it is equal to 0.706. According to the definition of Abundance-features, they can obviously be interpreted as indicators of cis-regulatory modules. Therefore, one can conclude that almost all TFs involved in the most important features prefer to bind to putative cis-regulatory modules. For example, according to the information for the important feature HEY1[501, 1000] in Table 7, we can expect the existence of a putative cis-regulatory module within [501, 1000] promoter regions of some genes, and this module contains HEY1, ZNF205, and IRF2. According to the information for TAF1[1, 100], another putative cis-regulatory module within [1, 100] promoter regions contains TAF1, NONO, CEBPD, and HEY1. On the one hand, HEY1, TAF1 and NONO are involved in the most important features. On the other hand, ZNF205, IRF2 and CEBPD are not directly involved and, possibly, can be classified as less important. Nevertheless, their impact on TSS activity was also taken into account because they participated in the selected ‘Abundance[-100, 0]’ feature. Thus, from the point of view of regression models, the most important features were related to TSS activity directly and individually while less important features were related with TSS activity mutually.

**Table 7:** Features that most correlate with the individual most important features identified for the HepG2 cell line.

Most important features	Feature_1	Corre-lation_1	Feature_2	Corre-lation_2	Feature_3	Corre-lation_3	Feature_4	Corre-lation_4	Feature_5	Corre-lation_5
HEY1[1, 100]	Abundance[1, 100]	0.657	Abundance[-100, 0]	0.624	HEY1[101, 500]	0.575	Abundance[101, 500]	0.552	NONO[1, 100]	0.548
HEY1[501, 1000]	Abundance[501, 1000]	0.689	HEY1[101, 500]	0.670	ZNF205[501, 1000]	0.590	IRF2[501, 1000]	0.590	Abundance[101, 500]	0.590
Abundance[-100, 0]	Abundance[1, 100]	0.787	ZNF205[-100, 0]	0.775	Abundance[-200, -101]	0.769	PPARG[-100, 0]	0.745	GATAD1[-100, 0]	0.744
TAF1[1, 100]	Abundance[1, 100]	0.617	NONO[1, 100]	0.595	Abundance[-100, 0]	0.524	CEBPD[1, 100]	0.514	HEY1[1, 100]	0.506
NONO[1, 100]	Abundance[1, 100]	0.622	TAF1[1, 100]	0.595	Abundance[-100, 0]	0.574	HEY1[1, 100]	0.548	CEBPD[1, 100]	0.527
HEY1[-1000, -501]	Abundance[-1000, -501]	0.706	HEY1[-500, -201]	0.634	ZNF205[-1000, -501]	0.613	CREB1[-1000, -501]	0.598	IRF2[-1000, -501]	0.591
AhR[-100, 0]	AhR[1, 100]	0.777	AhR[-200, -101]	0.766	AhR[101, 500]	0.613	AhR[-500, -201]	0.600	Abundance[-100, 0]	0.557
Sp1[-200, -101]	Abundance[-200, -101]	0.536	NF-YC[-200, -101]	0.445	CREM[-200, -101]	0.429	Abundance[-100, 0]	0.409	ATF1[-200, -101]	0.397
TBP[-100, 0]	Abundance[-100, 0]	0.635	Abundance[-200, -101]	0.531	Abundance[1, 100]	0.530	ZNF205[-100, 0]	0.519	TBP[1, 100]	0.508
c-Myc[-100, 0]	Abundance[-100, 0]	0.515	MAX[-100, 0]	0.438	HBP1[-100, 0]	0.410	TGIF2[-100, 0]	0.409	ZHX2[-100, 0]	0.404
HEY1[-5000, -1001]	Abundance[-5000, -1001]	0.647	MYBL2[-5000, -1001]	0.582	ERF[-5000, -1001]	0.567	ZNF205[-5000, -1001]	0.566	GATAD1[-5000, -1001]	0.564
NF-YC[501, 1000]	Abundance[501, 1000]	0.425	KLF10[501, 1000]	0.423	GMEB2[501, 1000]	0.415	RFXANK[501, 1000]	0.412	RFX5[501, 1000]	0.398
GR[-500, -201]	GR[-1000, -501]	0.467	GR[-200, -101]	0.435	TP53[-500, -201]	0.421	TP53[-1000, -501]	0.399	GR[101, 500]	0.391
HEY1[-100, 0]	HEY1[-500, -201]	0.421	HEY1[101, 500]	0.417	Abundance[-100, 0]	0.397	Abundance[-200, -101]	0.397	HEY1[-200, -101]	0.380
TAF1[-100, 0]	Abundance[-100, 0]	0.549	Abundance[-200, -101]	0.461	HBP1[-100, 0]	0.457	HMGXB4[-100, 0]	0.451	TBP[-100, 0]	0.447
TAF1[-1000, -501]	NONO[-1000, -501]	0.650	Abundance[-1000, -501]	0.628	TBP[-1000, -501]	0.585	KLF6[-1000, -501]	0.584	DMAP1[-1000, -501]	0.582
HEY1[-500, -201]	Abundance[-500, -201]	0.725	HEY1[-1000, -501]	0.634	ZNF205[-500, -201]	0.631	Abundance[-200, -101]	0.624	CREB1[-500, -201]	0.605
HNF3G[101, 500]	Abundance[101, 500]	0.541	NFIL3[101, 500]	0.461	RARA[101, 500]	0.455	NR2F6[101, 500]	0.442	MIER3[101, 500]	0.433
SSRP1[101, 500]	SSRP1[1, 100]	0.584	SSRP1[501, 1000]	0.490	SSRP1[-100, 0]	0.398	Abundance[101, 500]	0.348	PHF5A[101, 500]	0.336

According to the signs of the advanced regression coefficients, the most important features identified by advanced regressions can also be classified as activators or repressors. In particular, based on Table 6, one can conclude that five features (namely, HEY1 [-100, 0], AhR [-100, 0], NF-YC [501, 1000], TAF1 [-1000, -501] and HNF3G [101, 500]) can be classified as repressors, while the remaining features–as activators. However, one can expect that this classification can be distorted by the presence of Abundance-features among the most important features, since Abundance-features include information about many TFs. To understand how reliably stepwise regression models can actually classify features into activators or repressors, we conducted the following test. We removed all Abundance-features from the most important features and built the ordinary least squares regression models using the remaining important features. As a result, we observed a slight decrease in the accuracy of the regression but the regression coefficients and their significance were changed imperceptibly. Thus, in the case of the HepG2 cell line, the removal of the feature Abundance [-100, 0] resulted in a slight decrease in the *R*_o-p_ correlation coefficient from 0.743 to 0.739. However, the same five features can be classified as repressors due to the negative signs of their regression coefficients: HEY1 [-100, 0] (regression coefficient = -0.087, p-value = 1.512 × 10^−86^), AhR [-100, 0] (-0.048, 1.117 × 10^−78^), NF-YC [501, 1000], (-0.171, 2.736 × 10^−78^), TAF1 [-1000, -501] (-0.093, 9.954 × 10^−91^) and HNF3G [101, 500] (-0.087, 1.512 × 10^−86^). Thus, the presence of Abundance-features had no essential influence on repressor/activator classification.

**Fig 3:**
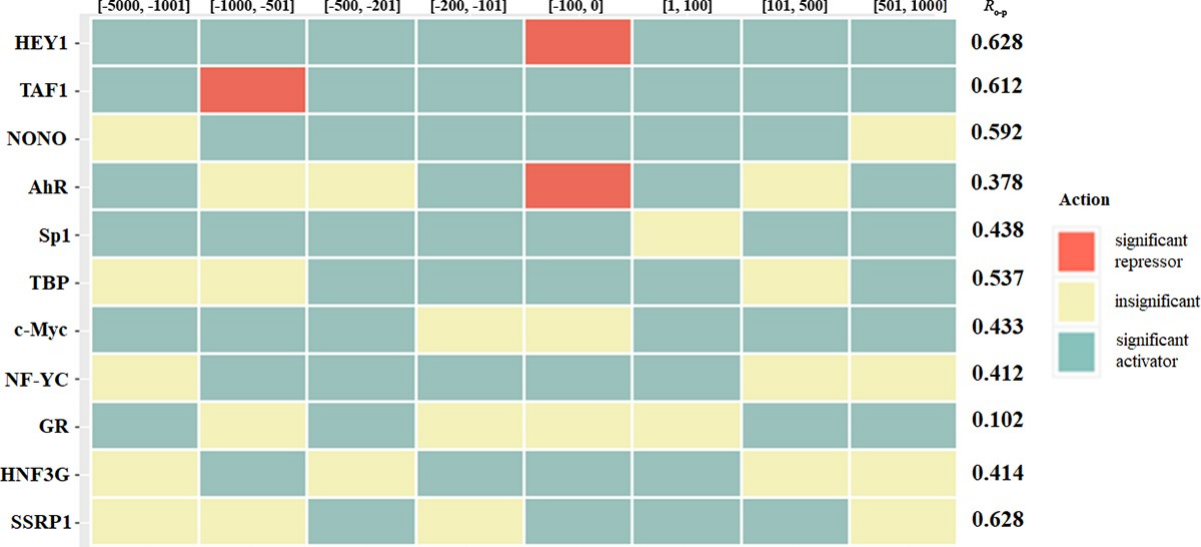
Classification of repressors/activators obtained by ordinary regressions using eight features for the HepG2 cell line.

It is interesting to note that we also considered an additional way of repressor/activator classification. In this case, each TF was analyzed independently. For each TF, we constructed the ordinary least squares regression model for which only eight binary features were used. Based on the signs of the regression coefficients and the p-values, we considered the following three categories: TF was classified as a significant repressor in a given promoter region if the sign of the regression coefficient of the corresponding feature was negative and p-value < 10^−5^. If the sign was positive and the p-value < 10^−5^, then TF was classified as a significant activator. If the p-value > 10^−5^, then TF was considered insignificant. Fig 3 shows the results of this classification for all 11 TFs, which were selected as the most important for the HepG2 cell line. The new classification approach confirmed most of the features from Table 6. Only three features (namely, c-Myc[-100, 0], NF-YC[501, 1000] and HNF3G[101, 500]) were classified as insignificant. However, it is necessary to note that the results of the new classification are less reliable, as the accuracy of ordinary least squares regressions was quite moderate, since *R*_o-p_ varied in the range [0.102, 0.628], see Fig 3.

On the one hand, for construction of regression models it is sufficient to use the ‘predicted mean profile’ and approximately 20 the most important features due to small increments of *R*_o-p_ in last steps of feature selection. According to their p-values, selected features are extremely significant. On the other hand, it seems likely that these sets of features can be extended by additionally composed the lists of attendant features that also play a role in cell-specific regulation. For this purpose, we continued to select features with the help of stepwise forward regression. In this case, we stopped selection when the p-value of least significant regression coefficient exceeded the threshold 10–20. The lists of attendant features for HepG2, K562 and HEK293 cell lines are available as S6 to S8 Tables, respectively. The attendant features can be classified as less important, but still highly significant for cell-specific regulation.

Additionally, we briefly considered the possibility of using advanced regression models obtained for one cell line (for example, HepG2) to predict TSS activities in another highly correlated cells (for example, primary hepatocytes). Unfortunately, this approach is not applicable (at least, intensively) to our features due to frequent incompleteness of the ChIP-Seq data. This incompleteness is due to the fact that, for example, for the cell line, the ChIP-Seq experiments were carried out using one set of TFs, while for the primary cells, the experiments were carried out with a different set of TFs. In particular, according to Table 6, to predict TSS activities in hepatocytes using the advanced regression model, it is necessary to have TFBRs of selected eleven TFs, while currently (according to the GTRD database) only CTCF, EZH2 and NR1H4 have been studied in ChIP-Seq experiments. Therefore, the advanced regression model mentioned in Table 6 is currently still not useful for predicting TSS activities in hepatocytes.

Finally, to demonstrate the usefulness of the predicted mean profile, we performed a regression analysis of three rare cell lines DU145 (prostate carcinoma), THP-1 (acute monocytic leukemia) and U937 (adult acute monocytic leukemia). Only a few TFs were studied in ChIP-Seq experiments on these cell lines, see Table 8. It was therefore not surprising that the primary regression models could only achieve low accuracy: 0.426 ≤ *R*_o-p_ ≤ 0.538. However, the accuracy increased 1.30–1.57 times when we used the predicted mean profile to build advanced regression models, see Table 8. These models are available in S9 to S11 Tables. The significant increment of *R*_o-p_ values indicated that a non-cell-specific feature (the predicted mean profile) could compensate, at least in part, for the absence of a large number of features in poorly studied cell lines.

**Table 8:** Accuracy of the primary and advanced regression models for the DU145, THP-1 and U937 cell lines.

Cell line	Number of TFs	*R*_o-p_ for primary regression model	*R*_o-p_ for advanced regression model
DU145	6	0.538	0.699
THP-1	11	0.440	0.652
U937	5	0.426	0.667

### Sum-transformation of expression levels for closely spaced TSSs

One of the specific properties of TSSs in the FANTOM5 atlas was that many of them are located close to each other. In particular, 116,620 TSSs (55.6%) had other TSSs nearby at a distance of less than 100 bp. For such closely spaced TSSs, we replaced their individual expression levels with sums of their expression levels and then calculate LTE-levels for sums. For the remaining TSSs, which were separated by at least 100 bp, we did not change their individual LTE-levels. As a result, for the given cell line, we created a new transcription profile, say, sum-transformed profile.

To predict sum-transformed profile, we also applied stepwise forward regression to PRIMARY_FEATURES. In other words, we have constructed primary regression models for prediction of sum-transformed profiles. S12 to S14 Tables contain the resulting sum-transformed regression models. The first row of Table 9 contains the accuracy of sum-transformed regression models for the HepG2, K562 and HEK293 cell lines. Comparison of *R*_o-p_ values in the first row of Table 9 with *R*_o-p_ values achieved using primary regression models (see Table 3) indicated that the accuracy increased 1.073–1.15 times. Thus, the transition from transcription activity profiles to sum-transformed profiles has become the second way to increase the accuracy of regression models.

**Table 9:** *R*_o-p_ correlations and percentages of identical features for sum-transformed regression models for the HepG2, K562 and HEK293 cell lines.

Characteristics of sum-transformed regression models	HepG2	K562	HEK293
*R*_o-p_ correlation	0.781	0.768	0.721
Percentage of identical features	60%	60%	55%

Finally, it is interesting to note that the list of features selected by the sum-transformed regression models and the list of features selected by the primary regression models were significantly overlapped. For example, Table 2 and S12 Table contained 12 (60%) identical features. The second row of Table 9 contains the percentage of identical features for the three analyzed cell lines.

## Conclusions

1. Using the stepwise forward regression method, we identified the sets of the most important TFs that affect expression activity of TSSs in human cell lines such as HepG2, K562 and HEK293.
2. With the help of the constructed regression models, we demonstrated that some TFs can be classified simultaneously as repressors and activators depending on their location relative to TSS.
3. A comparative analysis of cell lines revealed high similarity between them. We expressed the commonality of cell lines using the novel feature ‘predicted mean profile’. We demonstrated that this feature is useful for improving the accuracy of regression models, as well as for analyzing rare cell lines.

## Supporting information

S1 Fig Relationships between the transcriptional activity profile for HepG2 and the profile for HEK293 (upper figure) and the profile for K562 (lower figure). (PDF)

S1 Table Primary regression model for the K562 cell line. (DOCX)

S2 Table Primary regression model for the HEK293 cell line. (DOCX)

S3 Table Primary regression model K562_List-40. (DOCX)

S4 Table Advanced regression model for the K562 cell line. (DOCX)

S5 Table Advanced regression model for the HEK293 cell line. (DOCX)

S6 Table List of attendant features that are significantly cell-specific for regulation of HepG2. (DOCX)

S7 Table List of attendant features that are significantly cell-specific for regulation of K562. (DOCX)

S8 Table List of attendant features that are significantly cell-specific for regulation of HEK293. (DOCX)

S9 Table Advanced regression model for the DU145 cell line. (DOCX)

S10 Table Advanced regression model for the THP-1 cell line. (DOCX)

S11 Table Advanced regression model for the U937 cell line. (DOCX)

S12 Table Sum-transformed regression model for the HepG2 cell line. (DOCX)

S13 Table Sum-transformed regression model for the K562 cell line. (DOCX)

S14 Table Sum-transformed regression model for the HEK293 cell line. (DOCX)
