# Supplementary material for: Assessment of transcriptional importance of cell line-specific features based on GTRD and FANTOM5 data
Source: PLoS One. 2020 Dec 21;15(12):e0243332. doi: 10.1371/journal.pone.0243332 (PMC7751965; doi:10.1371/journal.pone.0243332)
Supplement: S1 Fig — (PDF) [file pone.0243332.s001.pdf]

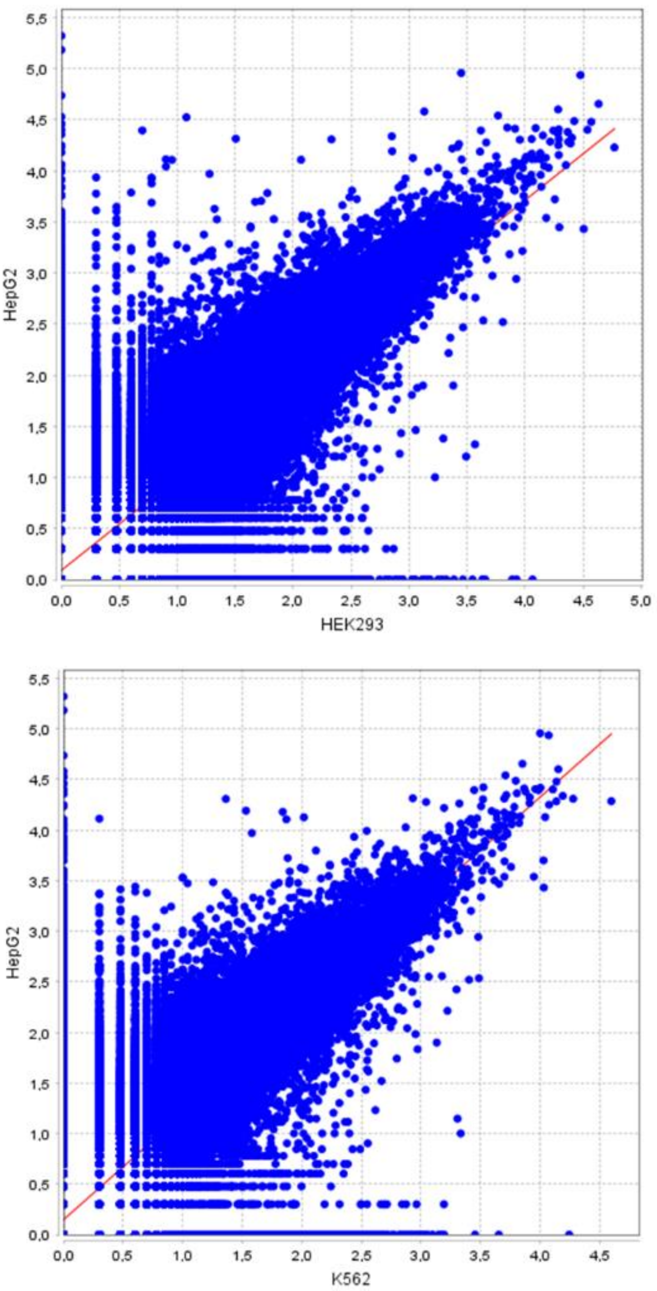

S1 Fig. Relationships between the transcriptional activity profile for HepG2 and the profile for HEK293 (upper figure, 1a) and the profile for K562 (lower figure, 1b).
