# Supplementary material for: Assessment of transcriptional importance of cell line-specific features based on GTRD and FANTOM5 data
Source: PLoS One. 2020 Dec 21;15(12):e0243332. doi: 10.1371/journal.pone.0243332 (PMC7751965; doi:10.1371/journal.pone.0243332)
Supplement: S1 Table — (DOCX) [file pone.0243332.s002.docx]

**S1 Table. Primary regression model for the K562 cell line.**

| **Feature** | **Correlation coefficient, R_o-p_** | **Increment of correlation coefficient** | **Regression coefficient** | **p-value** |
| --- | --- | --- | --- | --- |
| SMAD5 [1, 100] | 0.580 | 0.580 | 0.349 | < 1.0 × 10^-300^ |
| Abundance [-100, 0] | 0.625 | 0.045 | 0.467 | 9.540 × 10^-119^ |
| TAF1 [1, 100] | 0.640 | 0.015 | 0.136 | < 1.0 × 10^-300^ |
| NF-YA [-100, 0] | 0.651 | 0.011 | 0.162 | < 1.0 × 10^-300^ |
| JARID1B [1, 100] | 0.659 | 0.008 | 0.151 | < 1.0 × 10^-300^ |
| ZNF75A [-100, 0] | 0.667 | 0.008 | 0.245 | < 1.0 × 10^-300^ |
| c-Ets-1 [1, 100] | 0.673 | 0.006 | 0.194 | < 1.0 × 10^-300^ |
| SMAD1 [1, 100] | 0.678 | 0.005 | 0.138 | < 1.0 × 10^-300^ |
| Sp1 [-200, -101] | 0.681 | 0.003 | 0.117 | 2.747 × 10^-262^ |
| SMAD5 [-100, 0] | 0.684 | 0.003 | -0.141 | < 1.0 × 10^-300^ |
| ZFX [101, 500] | 0.687 | 0.003 | 0.108 | 4.207 × 10^-296^ |
| Abundance [1, 100] | 0.689 | 0.002 | -0.982 | < 1.0 × 10^-300^ |
| HEY1 [1, 100] | 0.692 | 0.003 | 0.109 | 1.447 × 10^-292^ |
| ZBED1 [1, 100] | 0.695 | 0.003 | 0.130 | 1.154 × 10^-234^ |
| MYC [-100, 0] | 0.697 | 0.002 | 0.094 | 3.919 × 10^-185^ |
| JARID1B [-100, 0] | 0.699 | 0.002 | -0.097 | 6.269 × 10^-204^ |
| HEY1 [501, 1000] | 0.701 | 0.002 | 0.109 | < 1.0 × 10^-300^ |
| ZNF639 [501, 1000] | 0.703 | 0.002 | -0.114 | 1.325 × 10^-192^ |
| NONO [1, 100] | 0.704 | 0.001 | 0.117 | 7.004 × 10^-181^ |
| SIX5 [-100, 0] | 0.704 | < 0.001 | 0.137 | 9.165 × 10^-158^ |
