# Supplementary material for: Assessment of transcriptional importance of cell line-specific features based on GTRD and FANTOM5 data
Source: PLoS One. 2020 Dec 21;15(12):e0243332. doi: 10.1371/journal.pone.0243332 (PMC7751965; doi:10.1371/journal.pone.0243332)
Supplement: S2 Table — (DOCX) [file pone.0243332.s003.docx]

**S2 Table. Primary regression model for the HEK293 cell line.**

| **Feature** | **Correlation coefficient, R_o-p_** | **Increment of correlation coefficient** | **Regression coefficient** | **p-value** |
| --- | --- | --- | --- | --- |
| Abundance [-100, 0] | 0.472 | 0.472 | -0.957 | 1.035 × 10^-206^ |
| MLL [101, 500] | 0.506 | 0.506 | 0.244 | < 1.0 × 10^-300^ |
| Sp2 [-100, 0] | 0.530 | 0.530 | 0.178 | < 1.0 × 10^-300^ |
| ELK4 [-100, 0] | 0.548 | 0.548 | 0.344 | < 1.0 × 10^-300^ |
| YY2 [1, 100] | 0.562 | 0.562 | 0.428 | < 1.0 × 10^-300^ |
| Abundance [-500, -201] | 0.573 | 0.573 | 0.912 | < 1.0 × 10^-300^ |
| ZSCAN22 [-100, 0] | 0.584 | 0.584 | 0.204 | < 1.0 × 10^-300^ |
| ZNF574 [-100, 0] | 0.590 | 0.590 | 0.207 | < 1.0 × 10^-300^ |
| ZBTB26 [101, 500] | 0.596 | 0.596 | 0.110 | 6.343 × 10^-209^ |
| KLF15 [-100, 0] | 0.601 | 0.601 | 0.153 | 1.817 × 10^-244^ |
| Sp3 [-200, -101] | 0.605 | 0.605 | 0.149 | 4.696 × 10^-296^ |
| ZNF518A [-100, 0] | 0.608 | 0.608 | 0.370 | < 1.0 × 10^-300^ |
| ZNF224 [101, 500] | 0.611 | 0.611 | 0.154 | 1.179 × 10^-200^ |
| MLL [-100, 0] | 0.614 | 0.614 | -0.235 | 9.480 × 10^-243^ |
| ZFP161 [-100, 0] | 0.616 | 0.616 | 0.143 | 1.171 × 10^-218^ |
| ZNF76 [-100, 0] | 0.619 | 0.619 | 0.156 | 4.718 × 10^-192^ |
| YY1 [1, 100] | 0.621 | 0.621 | 0.107 | < 1.0 × 10^-300^ |
| KLF12 [-100, 0] | 0.623 | 0.623 | 0.158 | 4.420 × 10^-167^ |
| ZXDB [101, 500] | 0.624 | 0.624 | 0.113 | 1.192 × 10^-178^ |
| ZNF384 [-100, 0] | 0.626 | 0.626 | 0.172 | 1.692 × 10^-241^ |
