# Supplementary material for: Assessment of transcriptional importance of cell line-specific features based on GTRD and FANTOM5 data
Source: PLoS One. 2020 Dec 21;15(12):e0243332. doi: 10.1371/journal.pone.0243332 (PMC7751965; doi:10.1371/journal.pone.0243332)
Supplement: S3 Table — (DOCX) [file pone.0243332.s004.docx]

**S3 Table. Primary regression model K562_List-40.**

| **Feature** | **Correlation coefficient, R_o-p_** | **Increment of correlation coefficient** | **Regression coefficient** | **p-value** |
| --- | --- | --- | --- | --- |
| GATA1[101, 500] | 0.408 | 0.408 | 0.088 | 8.112 × 10^-195^ |
| NF-YA [-100, 0] | 0.491 | 0.083 | 0.305 | < 1.0 × 10^-300^ |
| Sp1[-200, -101] | 0.526 | 0.035 | 0.183 | < 1.0 × 10^-300^ |
| SIX5[-100, 0] | 0.549 | 0.023 | 0.256 | < 1.0 × 10^-300^ |
| Mxi-1 [1, 100] | 0.565 | 0.016 | 0.217 | < 1.0 × 10^-300^ |
| GATA1 [501, 1000] | 0.576 | 0.011 | 0.119 | < 1.0 × 10^-300^ |
| GABP [-100, 0] | 0.584 | 0.008 | 0.103 | 2.340 × 10^-228^ |
| EGR1[-100,0] | 0.589 | 0.005 | 0.107 | 3.522 × 10^-260^ |
| BCL-3 [1, 100] | 0.594 | 0.005 | 0.250 | 1.218 × 10^-275^ |
| PU.1 [-100, 0] | 0.598 | 0.004 | 0.098 | 5.695 × 10^-224^ |
| Tal-1 [1, 100] | 0.601 | 0.003 | 0.151 | 2.699 × 10^-266^ |
| Sp2 [-100, 0] | 0.603 | 0.002 | 0.162 | 4.120 × 10^-171^ |
| GATA1[1, 100] | 0.605 | 0.002 | -0.091 | 4.941 × 10^-250^ |
| E2F-6 [101, 500] | 0.607 | 0.002 | 0.072 | 6.801 × 10^-181^ |
| NF-YA [1, 100] | 0.609 | 0.002 | -0.254 | 3.095 × 10^-226^ |
| GATA2 [101, 500] | 0.611 | 0.002 | -0.089 | 3.330 × 10^-218^ |
| ZBTB33 [-5000, -1001] | 0.613 | 0.002 | 0.046 | 5.771 × 10^-164^ |
| C/EBPδ [-500, -201] | 0.615 | 0.002 | 0.064 | 9.701 × 10^-95^ |
| THAP1[-100, 0] | 0.616 | 0.001 | 0.140 | 1.565 × 10^-104^ |
| NF-YA [-200, -101] | 0.617 | 0.001 | 0.092 | 1.652 × 10^-91^ |
