# Supplementary material for: Assessment of transcriptional importance of cell line-specific features based on GTRD and FANTOM5 data
Source: PLoS One. 2020 Dec 21;15(12):e0243332. doi: 10.1371/journal.pone.0243332 (PMC7751965; doi:10.1371/journal.pone.0243332)
Supplement: S4 Table — (DOCX) [file pone.0243332.s005.docx]

**S4 Table. Advanced regression model for the K562 cell line.**

| **Feature** | **Correlation coefficient, R_o-p_** | **Increment of correlation coefficient** | **Regression coefficient** | **p-value** |
| --- | --- | --- | --- | --- |
| Predicted mean profile | 0.707 | 0.707 | 0.681 | < 1.0 × 10^-300^ |
| SMAD5 [1, 100] | 0.716 | 0.009 | 0.203 | < 1.0 × 10^-300^ |
| TAF1 [1, 100] | 0.718 | 0.002 | 0.104 | 9.942 × 10^-225^ |
| ZBED1 [-100, 0] | 0.720 | 0.002 | 0.097 | 2.453 × 10^-161^ |
| SMAD5 [-100, 0] | 0.721 | 0.001 | -0.089 | 2.667 × 10^-157^ |
| MYC [-100, 0] | 0.722 | 0.001 | 0.088 | 6.454 × 10^-199^ |
| CTCFL [101, 500] | 0.723 | 0.001 | -0.043 | 4.269 × 10^-88^ |
| HEY1 [501, 1000] | 0.724 | 0.001 | 0.064 | 2.110 × 10^-127^ |
| ERG [-500, -201] | 0.725 | 0.001 | -0.061 | 6.830 × 10^-108^ |
| ZNF592 [-500, -201] | 0.726 | 0.001 | 0.068 | 1.089 × 10^-132^ |
| HEY1 [-100, 0] | 0.727 | 0.001 | -0.065 | 2.758 × 10^-121^ |
| ZNF639 [501, 1000] | 0.727 | < 0.001 | -0.074 | 3.592 × 10^-80^ |
| GATA1 [-100, 0] | 0.728 | 0.001 | -0.046 | 1.234 × 10^-92^ |
| HEY1 [1, 100] | 0.728 | < 0.001 | 0.067 | 1.837 × 10^-119^ |
| Mxi1 [1, 100] | 0.729 | 0.001 | 0.102 | 3.516 × 10^-135^ |
| NFATc3 [-100, 0] | 0.729 | < 0.001 | 0.133 | 7.939 × 10^-102^ |
| Abundance [1, 100] | 0.730 | 0.001 | -0.475 | 1.522 × 10^-157^ |
| SMAD1 [1, 100] | 0.731 | 0.001 | 0.070 | 4.892 × 10^-101^ |
| Mnt [501, 1000] | 0.732 | 0.001 | 0.057 | 2.305 × 10^-89^ |
| Sp1 [501, 1000] | 0.732 | < 0.001 | -0.101 | 1.785 × 10^-73^ |
