# Supplementary material for: Assessment of transcriptional importance of cell line-specific features based on GTRD and FANTOM5 data
Source: PLoS One. 2020 Dec 21;15(12):e0243332. doi: 10.1371/journal.pone.0243332 (PMC7751965; doi:10.1371/journal.pone.0243332)
Supplement: S5 Table — (DOCX) [file pone.0243332.s006.docx]

**S5 Table. Advanced regression model for the HEK293 cell line.**

| **Feature** | **Correlation coefficient, R_o-p_** | **Increment of correlation coefficient** | **Regression coefficient** | **p-value** |
| --- | --- | --- | --- | --- |
| Predicted mean profile | 0.723 | 0.723 | 1.033 | < 1.0 × 10^-300^ |
| Abundance [101, 500] | 0.725 | 0.002 | 0.218 | 7.658 × 10^-40^ |
| ZSCAN22 | 0.726 | 0.001 | 0.064 | 3.283 × 10^-72^ |
| MLL [501, 1000] | 0.727 | 0.001 | 0.077 | 2.769 × 10^-70^ |
| ZNF574 [-100, 0] | 0.728 | 0.001 | 0.064 | 5.011 × 10^-71^ |
| ZNF335 [-500, -201] | 0.728 | < 0.001 | 0.054 | 4.326 × 10^-78^ |
| ZNF335 [-100, 0] | 0.729 | 0.001 | -0.051 | 3.740 × 10^-47^ |
| Sp2 [-100, 0] | 0.729 | < 0.001 | 0.051 | 1.118 × 10^-61^ |
| ZFP161 [-1000, -501] | 0.729 | < 0.001 | -0.062 | 3.283 × 10^-47^ |
| IKZF3 [-200, -101] | 0.730 | 0.001 | 0.058 | 3.88 × 10^-40^ |
| KLF15 [501, 1000] | 0.730 | < 0.001 | -0.049 | 1.198 × 10^-27^ |
| ELK4 [-100, 0] | 0.730 | < 0.001 | 0.080 | 1.202 × 10^-39^ |
| 2.3.3.0.1 [1, 100] | 0.730 | < 0.001 | 0.050 | 1.968 × 10^-35^ |
| MLL [-100, 0] | 0.731 | 0.001 | -0.085 | 1.75 × 10^-43^ |
| MLL [101, 500] | 0.731 | < 0.001 | 0.058 | 5.754 × 10^-46^ |
| ZFP64 [-100, 0] | 0.731 | < 0.001 | -0.036 | 3.576 × 10^-31^ |
| ZNF362 [-100, 0] | 0.731 | < 0.001 | 0.095 | 1.944 × 10^-28^ |
| Sp4 [-200, -101] | 0.731 | < 0.001 | 0.039 | 1.605 × 10^-29^ |
| ZXDB [1, 100] | 0.732 | 0.001 | 0.049 | 5.508 × 10^-37^ |
| ZNF600 [-100, 0] | 0.732 | < 0.001 | -0.037 | 9.724 × 10^-40^ |
