# Supplementary material for: Assessment of transcriptional importance of cell line-specific features based on GTRD and FANTOM5 data
Source: PLoS One. 2020 Dec 21;15(12):e0243332. doi: 10.1371/journal.pone.0243332 (PMC7751965; doi:10.1371/journal.pone.0243332)
Supplement: S6 Table — (DOCX) [file pone.0243332.s007.docx]

**S6 Table. List of attendant features that are significantly cell-specific for regulation of HepG2.**

| **Feature** | **p-value** |
| --- | --- |
| C/EBPδ [1, 100] | 9.616 × 10^-54^ |
| HNF-4γ [-100, 0] | 8.668 × 10^-61^ |
| ZNF76 [1, 100] | 3.774 × 10^-52^ |
| JARID1A [1, 100] | 2.692 × 10^-97^ |
| FOXO3 [1, 100] | 1.338 × 10^-70^ |
| TAF-1 [101, 500] | 2.029 × 10^-43^ |
| p66-α [1, 100] | 1.871 × 10^-31^ |
| GR [501, 1000] | 2.334 × 10^-46^ |
| GATA-4 [1, 100] | 7.709 × 10^-35^ |
| RFX [101, 500] | 1.108 × 10^-40^ |
| MYST2 [501, 1000] | 3.341 × 10^-82^ |
| HNF3G [501, 1000] | 9.522 × 10^-54^ |
| JARID1 [-100, 0] | 1.244 × 10^-42^ |
| ZSCAN2 [-200, -101] | 2.306 × 10^-40^ |
| c-Jun [-100, 0] | 4.636 × 10^-35^ |
| ZBTB7A [-1000, -501] | 4.916 × 10^-23^ |
| TEF-3 [-500, -201] | 9.027 × 10^-55^ |
| CTCF [1, 100] | 9.981 × 10^-25^ |
| YY1 [1, 100] | 3.796 × 10^-37^ |
| T3R-β [1, 100] | 1.791 × 10^-26^ |
| Mad3 [-100, 0] | 2.355 × 10^-25^ |
| THAP11 [-100, 0]] | 1.430 × 10^-32^ |
| SSRP1 [501, 1000] | 3.363 × 10^-44^ |
| c-Ets-1 [501, 1000] | 1.024 × 10^-23^ |
| JARID1A [101, 500] | 9.113 × 10^-30^ |
| GMEB-2 [1, 100] | 3.411 × 10^-27^ |
| FOXO3 [-100, 0] | 1.551 × 10^-25^ |
| RCoR2 [-500, -201] | 1.598 × 10^-26^ |
| RAR-α [-500, -201] | 1.062 × 10^-31^ |
| PHF5A [-500, -201] | 5.459 × 10^-27^ |
| ARID2 [-100, 0] | 2.715 × 10^-22^ |
| Sp5 [-100, 0] | 1.977 × 10^-22^ |
| Fra-2 [1, 100] | 1.847 × 10^-27^ |
| TGIF-2 [1, 100] | 7.543 × 10^-21^ |
| KLF16 [501, 1000] | 1.992 × 10^-39^ |
| ZHX2 [501, 1000] | 1.052 × 10^-22^ |
