# Supplementary material for: Assessment of transcriptional importance of cell line-specific features based on GTRD and FANTOM5 data
Source: PLoS One. 2020 Dec 21;15(12):e0243332. doi: 10.1371/journal.pone.0243332 (PMC7751965; doi:10.1371/journal.pone.0243332)
Supplement: S7 Table — (DOCX) [file pone.0243332.s008.docx]

**S7 Table. List of attendant features that are significantly cell-specific for regulation of K562.**

| **Feature** | **p-value** |
| --- | --- |
| c-Myc [101, 500] | 3.382 × 10^-79^ |
| C/EBPδ [1, 100] | 3.339 × 10^-78^ |
| C/EBPδ [ 101, 500] | 3.198 × 10^-43^ |
| SMAD5 [101, 500] | 2.886 × 10^-77^ |
| ZBTB33 [501, 1000] | 1.344 × 10^-49^ |
| ARID2 [501, 1000] | 4.307 × 10^-56^ |
| Arnt [-500, -201] | 3.575 × 10^-38^ |
| SMARCE1 [-100, 0] | 2.644 × 10^-81^ |
| NCoA-1 [101, 500] | 2.266 × 10^-41^ |
| SMARCE1 [501, 1000] | 1.368 × 10^-46^ |
| GATA-2 [501, 1000] | 2.139 × 10^-40^ |
| GATA-2 [-1000, -501] | 7.597 × 10^-38^ |
| ZNF75A [-100, 0] | 1.993 × 10^-43^ |
| SMAD5 [501, 1000] | 8.172 × 10^-65^ |
| Abundance [501, 1000] | 2.260 × 10^-58^ |
| Mxi-1 [501, 1000] | 6.053 × 10^-54^ |
| VEZF1 [-100, 0] | 8.411 × 10^-41^ |
| ZNF592 [-5000, -1001] | 5.257 × 10^-37^ |
| ZNF282 [-100, 0] | 1.822 × 10^-32^ |
| c-Ets-1 [1, 100] | 6.980 × 10^-29^ |
| JARID1B [1, 100] | 5.151 × 10^-47^ |
| ZFX [-100, 0] | 1.532 × 10^-27^ |
| KLF16 [1, 100] | 6.665 × 10^-36^ |
| FOXM1 [-200, -101] | 8.747 × 10^-29^ |
| NCoA-1 [501, 1000] | 2.996 × 10^-37^ |
| ZNF18 [1, 100] | 1.383 × 10^-28^ |
| JARID1B [101, 500] | 3.411 × 10^-28^ |
| YY1 [1, 100] | 5.616 × 10^-28^ |
| CREB-3 [101, 500] | 1.689 × 10^-24^ |
| Elf-4 [1, 100] | 5.211 × 10^-25^ |
| ZNF3 [101, 500] | 4.870 × 10^-23^ |
| MTA3 [1, 100] | 1.653 × 10^-24^ |
| CREM [-100, 0] | 3.803 × 10^-22^ |
| ZNF263 [-100, 0] | 3.644 × 10^-21^ |
