# Supplementary material for: Assessment of transcriptional importance of cell line-specific features based on GTRD and FANTOM5 data
Source: PLoS One. 2020 Dec 21;15(12):e0243332. doi: 10.1371/journal.pone.0243332 (PMC7751965; doi:10.1371/journal.pone.0243332)
Supplement: S8 Table — (DOCX) [file pone.0243332.s009.docx]

**S8 Table. List of attendant features that are significantly cell-specific for regulation of HEK293.**

| **Feature** | **p-value** |
| --- | --- |
| ZNF580 [-200, -101] | 1.909 × 10^-28^ |
| ZNF423 [1, 100] | 2.393 × 10^-24^ |
| ZNF35 [1, 100] | 3.608 × 10^-23^ |
| ZNF224 [101, 500] | 4.263 × 10^-23^ |
| YY2 [-5000, -1001] | 1.683 × 10^-21^ |
| KLF1 [1, 100] | 4.442 × 10^-26^ |
| Sp4 [-100, 0] | 4.725 × 10^-44^ |
| Sp1_-100_0 | 1.933 × 10^-31^ |
| ZNF554 [101, 500] | 2.249 × 10^-25^ |
| ZNF600 [4, 501, 1000] | 6.311 × 10^-27^ |
| KLF17 [-100, 0] | 7.943 × 10^-41^ |
| ZNF518A [-100, 0] | 4.562 × 10^-24^ |
| KLF17 [1, 100] | 1.954 × 10^-21^ |
| ZNF561 [501, 1000] | 6.992 × 10^-23^ |
| ZFP161 [-100, 0] | 2.401 × 10^-22^ |
| YY2 [101, 500] | 1.638 × 10^-21^ |
| ZIC2 [-5000, -1001] | 1.804 × 10^-46^ |
| ZSCAN30 [-5000, -1001] | 6.593 × 10^-30^ |
