# Supplementary material for: Assessment of transcriptional importance of cell line-specific features based on GTRD and FANTOM5 data
Source: PLoS One. 2020 Dec 21;15(12):e0243332. doi: 10.1371/journal.pone.0243332 (PMC7751965; doi:10.1371/journal.pone.0243332)
Supplement: S9 Table — (DOCX) [file pone.0243332.s010.docx]

**S9 Table. Advanced regression model for the DU145 cell line.**

| **Feature** | **Correlation coefficient, R_o-p_** | **Increment of correlation coefficient** | **Regression coefficient** | **p-value** |
| --- | --- | --- | --- | --- |
| Predicted mean profile | 0.689 | 0.689 | 1.026 | < 1.0 × 10^-300^ |
| c-Ets-1 [1, 100] | 0.695 | 0.006 | 0.099 | 4.303 × 10^-117^ |
| c-Ets-1 [101, 500] | 0.696 | 0.001 | 0.072 | 2.489 × 10^-70^ |
| Abundance [-500, -201] | 0.697 | 0.001 | 0.081 | 9.407 × 10^-8^ |
| Abundance [-5000, -1001 | 0.698 | 0.001 | 0.085 | 4.040 × 10^-16^ |
| Abundance [-100, 0] | 0.698 | < 0.001 | 0.192 | 1.635 × 10^-21^ |
| JARID1D [-1000, -501] | 0.698 | < 0.001 | 0.040 | 5.047 × 10^-16^ |
| GABPα [501, 1000] | 0.698 | < 0.001 | -0.108 | 2.015 × 10^-15^ |
| GABPα [-100, 0] | 0.699 | 0.001 | 0.083 | 9.258 × 10^-25^ |
| GABPα [-200, -101] | 0.699 | < 0.001 | -0.078 | 8.382 × 10^-18^ |
| GABPα [1, 100] | 0.699 | < 0.001 | -0.061 | 4.306 × 10^-8^ |
| FOXA1 [1, 100] | 0.699 | < 0.001 | -0.084 | 4.177 × 10^-11^ |
| c-Ets-1 [501, 1000] | 0.699 | < 0.001 | 0.024 | 3.957 × 10^-10^ |
| JARID1D [-500, -201] | 0.699 | < 0.001 | 0.035 | 4.093 × 10^-11^ |
| JARID1D [-100, 0] | 0.699 | < 0.001 | -0.041 | 1.577 × 10^-10^ |
| JunD [101, 500] | 0.699 | < 0.001 | 0.069 | 4.525 × 10^-9^ |
| GABPα [101, 500] | 0.699 | < 0.001 | -0.056 | 1.264 × 10^-8^ |
| FOXA1 [501, 1000] | 0.699 | < 0.001 | -0.054 | 2.213 × 10^-6^ |
| JARID1D [-5000, -1001] | 0.699 | < 0.001 | 0.022 | 3.435 × 10^-6^ |
| Abundance [-200, -101] | 0.699 | < 0.001 | 0.081 | 8.472 × 10^-6^ |
