# Supplementary material for: Assessment of transcriptional importance of cell line-specific features based on GTRD and FANTOM5 data
Source: PLoS One. 2020 Dec 21;15(12):e0243332. doi: 10.1371/journal.pone.0243332 (PMC7751965; doi:10.1371/journal.pone.0243332)
Supplement: S10 Table — (DOCX) [file pone.0243332.s011.docx]

**S10 Table. Advanced regression model for the THP-1 cell line.**

| **Feature** | **Correlation coefficient, R_o-p_** | **Increment of correlation coefficient** | **Regression coefficient** | **p-value** |
| --- | --- | --- | --- | --- |
| Predicted mean profile | 0.646 | 0.646 | 0.762 | < 1.0 × 10^-300^ |
| Abundance [-100, 0] | 0.648 | 0.002 | 0.166 | 9.196 × 10^-35^ |
| VDR [501, 1000] | 0.649 | 0.001 | 0.051 | 9.467 × 10^-95^ |
| Runx1 [1, 100] | 0.649 | < 0.001 | 0.040 | 6.948 × 10^-50^ |
| Intercept | 0.650 | 0.001 | -0.051 | < 1.0 × 10^-300^ |
| RCoR1 [-100, 0] | 0.650 | < 0.001 | -0.046 | 1.081 × 10^-24^ |
| MLL [501, 1000] | 0.650 | < 0.001 | 0.0218 | 3.644 × 10^-20^ |
| MLL [-100, 0] | 0.651 | 0.001 | -0.031 | 4.808 × 10^-17^ |
| Runx1 [501, 1000] | 0.651 | < 0.001 | 0.027 | 2.996 × 10^-23^ |
| MLL2 [501, 1000] | 0.651 | < 0.001 | -0.029 | 2.772 × 10^-21^ |
| PU.1 [1, 100] | 0.651 | < 0.001 | -0.027 | 1.039 × 10^-21^ |
| PU.1 [-100, 0] | 0.651 | < 0.001 | 0.034 | 1.826 × 10^-28^ |
| VDR [-500, -201] | 0.651 | < 0.001 | 0.019 | 1.172 × 10^-12^ |
| PU.1 [-200, -101] | 0.651 | < 0.001 | -0.016 | 3.140 × 10^-10^ |
| CTCF [1, 100] | 0.651 | < 0.001 | -0.034 | 2.162 × 10^-11^ |
| VDR [-5000, -1001] | 0.652 | 0.001 | 0.011 | 3.687 × 10^-10^ |
| Runx1 [-200, -101] | 0.652 | < 0.001 | 0.016 | 1.089 × 10^-9^ |
| MLL [1, 100] | 0.652 | 0.652 | 0.018 | 7.333 × 10^-8^ |
| PU.1 [-500, -201] | 0.652 | 0.652 | -0.013 | 2.073 × 10^-8^ |
| RCoR1 [-1000, -501] | 0.652 | 0.652 | 0.025 | 4.660 × 10^-8^ |
