# Supplementary material for: Assessment of transcriptional importance of cell line-specific features based on GTRD and FANTOM5 data
Source: PLoS One. 2020 Dec 21;15(12):e0243332. doi: 10.1371/journal.pone.0243332 (PMC7751965; doi:10.1371/journal.pone.0243332)
Supplement: S11 Table — (DOCX) [file pone.0243332.s012.docx]

**S11 Table. Advanced regression model for the U937 cell line.**

| **Feature** | **Correlation coefficient, R_o-p_** | **Increment of correlation coefficient** | **Regression coefficient** | **p-value** |
| --- | --- | --- | --- | --- |
| Predicted mean profile | 0.660 | 0.660 | 0.951 | < 1.0 × 10^-300^ |
| Runx1 [-100, 0] | 0.662 | 0.002 | 0.128 | 2.554 × 10^-87^ |
| C/EBPα [-100, 0] | 0.663 | 0.001 | 0.059 | 9.576 × 10^-70^ |
| C/EBPα [-5000, -1001] | 0.663 | < 0.001 | 0.029 | 8.129 × 10^-56^ |
| EGR [-500, -201] | 0.664 | 0.001 | -0.056 | 4.707 × 10^-58^ |
| Abundance [-500, -201] | 0.664 | < 0.001 | 0.118 | 1.323 × 10^-29^ |
| EGR [501, 1000] | 0.664 | < 0.001 | -0.033 | 6.539 × 10^-43^ |
| C/EBPα [501, 1000] | 0.665 | 0.001 | 0.039 | 2.554 × 10^-37^ |
| Intercept | 0.665 | < 0.001 | -0.031 | 1.982 × 10^-5^ |
| MIXL1 [101, 500] | 0.665 | < 0.001 | 0.045 | 1.895 × 10^-33^ |
| ZNF143 [-100, 0] | 0.666 | 0.001 | 0.099 | 4.417 × 10^-36^ |
| C/EBPα [-1000, -501] | 0.666 | < 0.001 | 0.029 | 1.031 × 10^-22^ |
| ZNF143 [1, 100] | 0.667 | 0.001 | -0.110 | 2.649 × 10^-15^ |
| MIXL1 [-5000, -1001] | 0.667 | < 0.001 | 0.016 | 5.288 × 10^-12^ |
| EGR [-1000, -501] | 0.667 | < 0.001 | -0.015 | 9.314 × 10^-9^ |
