# Supplementary material for: Assessment of transcriptional importance of cell line-specific features based on GTRD and FANTOM5 data
Source: PLoS One. 2020 Dec 21;15(12):e0243332. doi: 10.1371/journal.pone.0243332 (PMC7751965; doi:10.1371/journal.pone.0243332)
Supplement: S12 Table — (DOCX) [file pone.0243332.s013.docx]

**S12 Table. Sum-transformed regression model for the HepG2 cell line.**

| **Feature** | **Correlation coefficient, R_o-p_** | **Increment of correlation coefficient** | **Regression coefficient** | **p-value** |
| --- | --- | --- | --- | --- |
| Abundance [1, 100] | 0.652 | 0.652 | 0.712 | 5.360 × 10^-264^ |
| Abundance [-200, -101] | 0.693 | 0.041 | 0.871 | < 1.0 × 10^-300^ |
| TAF1 [1, 100] | 0.716 | 0.023 | 0.369 | < 1.0 × 10^-300^ |
| HEY1 [501, 1000] | 0.732 | 0.016 | 0.273 | < 1.0 × 10^-300^ |
| TAF1 [-100, 0] | 0.742 | 0.010 | 0.285 | < 1.0 × 10^-300^ |
| p53 [-1000, -501] | 0.749 | 0.007 | 1.084 | < 1.0 × 10^-300^ |
| NONO [1, 100] | 0.757 | 0.008 | 0.305 | < 1.0 × 10^-300^ |
| HEY1 [-500, -201] | 0.761 | 0.004 | 0.198 | < 1.0 × 10^-300^ |
| C/EBP δ [1, 100] | 0.764 | 0.003 | 0.189 | 2.283 × 10^-316^ |
| JARID1A [1, 100] | 0.766 | 0.002 | 0.141 | 1.776 × 10^-71^ |
| Sp1 [101, 500] | 0.769 | 0.003 | -0.370 | < 1.0 × 10^-300^ |
| HEY1 [101,500] | 0.771 | 0.002 | 0.189 | < 1.0 × 10^-300^ |
| GR [101, 500] | 0.773 | 0.002 | 1.220 | < 1.0 × 10^-300^ |
| KLF10 [-100, 0] | 0.775 | 0.002 | 0.188 | 3.466 × 10^-225^ |
| KLF10 [501, 1000] | 0.777 | 0.002 | -0.356 | < 1.0 × 10^-300^ |
| MYST2 [101, 500] | 0.778 | 0.001 | 0.155 | 3.757 × 10^-284^ |
| YY1 [1, 100] | 0.779 | 0.001 | 0.143 | 2.466 × 10^-180^ |
| TBP [-100, 0] | 0.780 | 0.001 | 0.142 | 2.497 × 10^-181^ |
| TEF1 [101, 500] | 0.781 | 0.001 | -0.123 | 6.518 × 10^-174^ |
| JARID1A [101, 500] | 0.781 | < 0.001 | 0.193 | 5.372 × 10^-163^ |
