# Supplementary material for: Assessment of transcriptional importance of cell line-specific features based on GTRD and FANTOM5 data
Source: PLoS One. 2020 Dec 21;15(12):e0243332. doi: 10.1371/journal.pone.0243332 (PMC7751965; doi:10.1371/journal.pone.0243332)
Supplement: S13 Table — (DOCX) [file pone.0243332.s014.docx]

**S13 Table. Sum-transformed regression model for the K562 cell line.**

| **Feature** | **Correlation coefficient, R_o-p_** | **Increment of correlation coefficient** | **Regression coefficient** | **p-value** |
| --- | --- | --- | --- | --- |
| Abundance [1, 100] | 0.619 | 0.619 | -0.302 | 1.388 × 10^-40^ |
| SMAD5 [1, 100] | 0.668 | 0.049 | 0.291 | < 1.0 × 10^-300^ |
| Abundance [-500, -201] | 0.690 | 0.022 | 0.637 | < 1.0 × 10^-300^ |
| ZFX [101, 500] | 0.705 | 0.015 | 0.227 | < 1.0 × 10^-300^ |
| SMAD1 [1, 100] | 0.715 | 0.010 | 0.204 | < 1.0 × 10^-300^ |
| Sp1 [-200, -101] | 0.723 | 0.008 | 0.201 | < 1.0 × 10^-300^ |
| ZNF75A [-100, 0] | 0.730 | 0.007 | 0.310 | < 1.0 × 10^-300^ |
| c-Ets-1 [1, 100] | 0.735 | 0.005 | 0.254 | < 1.0 × 10^-300^ |
| TAF1 [-100, 0] | 0.740 | 0.005 | 0.147 | < 1.0 × 10^-300^ |
| NF-YA [-100, 0] | 0.745 | 0.005 | 0.223 | < 1.0 × 10^-300^ |
| HEY1 [501, 1000] | 0.748 | 0.003 | 0.192 | < 1.0 × 10^-300^ |
| ZNF639 [501, 1000] | 0.752 | 0.004 | -0.204 | < 1.0 × 10^-300^ |
| ZNF410 [1, 100] | 0.755 | 0.003 | 0.712 | < 1.0 × 10^-300^ |
| NONO [1, 100] | 0.758 | 0.003 | 0.202 | < 1.0 × 10^-300^ |
| GATA1 [501, 1000] | 0.760 | 0.002 | 0.128 | < 1.0 × 10^-300^ |
| TAF1 [1, 100] | 0.762 | 0.002 | 0.165 | < 1.0 × 10^-300^ |
| POU5F1 [-200, -101] | 0.764 | 0.002 | 0.207 | 3.624 × 10^-284^ |
| MYC [-100, 0] | 0.766 | 0.002 | 0.134 | 4.141 × 10^-279^ |
| ZBED1 [1, 100] | 0.767 | 0.001 | 0.168 | 3.502 × 10^-259^ |
| NGFI-B [501, 1000] | 0.768 | 0.001 | -0.170 | 1.019 × 10^-259^ |
