# Supplementary material for: Assessment of transcriptional importance of cell line-specific features based on GTRD and FANTOM5 data
Source: PLoS One. 2020 Dec 21;15(12):e0243332. doi: 10.1371/journal.pone.0243332 (PMC7751965; doi:10.1371/journal.pone.0243332)
Supplement: S14 Table — (DOCX) [file pone.0243332.s015.docx]

**S14 Table. Sum-transformed regression model for the HEK293 cell line.**

| **Feature** | **Correlation coefficient, R_o-p_** | **Increment of correlation coefficient** | **Regression coefficient** | **p-value** |
| --- | --- | --- | --- | --- |
| Abundance [-100, 0] | 0.560 | 0.560 | -0.721 | 7.215 × 10^-66^ |
| MLL [101, 500] | 0.611 | 0.051 | 0.412 | < 1.0 × 10^-300^ |
| Abundance [-500, -201] | 0.640 | 0.029 | 1.603 | < 1.0 × 10^-300^ |
| ZBTB26 [101, 500] | 0.653 | 0.013 | 0.157 | 8.573 × 10^-284^ |
| ELK4 [-100, 0] | 0.666 | 0.013 | 0.435 | < 1.0 × 10^-300^ |
| ZSCAN22 [-100, 0] | 0.677 | 0.011 | 0.271 | < 1.0 × 10^-300^ |
| YY2 [1, 100] | 0.683 | 0.006 | 0.462 | < 1.0 × 10^-300^ |
| Sp2 [-200, -101] | 0.689 | 0.006 | 0.160 | 1.401 × 10^-296^ |
| Sp2 [-100, 0] | 0.694 | 0.005 | 0.194 | < 1.0 × 10^-300^ |
| ZNF574 [-100, 0] | 0.699 | 0.005 | 0.233 | < 1.0 × 10^-300^ |
| ZNF224 [101, 500] | 0.702 | 0.003 | 0.215 | 3.023 × 10^-275^ |
| KLF15 [-200, -101] | 0.706 | 0.004 | 0.213 | < 1.0 × 10^-300^ |
| Abundance [1,100] | 0.708 | 0.002 | 1.724 | < 1.0 × 10^-300^ |
| MLL [-200, -101] | 0.711 | 0.003 | -0.3102 | 9.365 × 10^-271^ |
| ZFP161 [-100, 0] | 0.713 | 0.002 | 0.198 | 4.210 × 10^-301^ |
| ZNF518A [-200, -101] | 0.715 | 0.002 | 0.401 | 3.627 × 10^-293^ |
| KLF1 [-100, 0] | 0.717 | 0.002 | 0.209 | 4.135 × 10^-228^ |
| ZNF175 [101, 500] | 0.719 | 0.002 | 0.571 | 8.141 × 10^-217^ |
| YY1 [101, 500] | 0.720 | 0.001 | 0.141 | < 1.0 × 10^-300^ |
| ZFP161 [-1000, -501] | 0.721 | 0.001 | -0.178 | 8.991 × 10^-201^ |
